# Changing the Climate in Information Systems Research

**DOI:** 10.1007/s12599-021-00695-y

**Published:** 2021-03-25

**Authors:** Sebastian Lehnhoff, Philipp Staudt, Richard T. Watson

**Affiliations:** 1 Department of Energy Informatics, University of Oldenburg, Oldenburg, Germany; 2 Karlsruhe Institute of Technology (KIT), Institute of Information Systems and Marketing (IISM), Karlsruhe, Germany; 3 Department of Management Information Systems, Terry College of Business, University of Georgia, Athens, GA USA

## Introduction

The Covid-19 pandemic has demonstrated society’s ability to adapt to a crisis and its ingenuity in developing solutions. Digitalization has played a major role in handling the virus. Conferencing tools have allowed for remote work and homeschooling (Hacker et al. 2020), mobile applications have helped to trace infections (Trang et al. 2020), and personal GPS data gave scientists the opportunity to create models of mobility patterns and evaluate the success of social distancing measures (Chang et al. 2021).

A similar, but at least an order of magnitude greater, effort is needed from Information Systems and Computer Science scholars to develop solutions that reduce carbon emissions to finally decelerate climate change effectively. Practitioners have shown a strong willingness to implement such solutions. In September 2020, Google pledged to run on carbon-free energy 24/7 by 2030, meaning that it will shift from meeting carbon reduction goals by buying renewable generation certificates to satisfying its energy demand through generated carbon-free electricity in real-time (Miller 2020). This is a major milestone as, currently, corresponding carbon-free energy products are not always available for all data centers. To achieve its goals, Google has identified options to run its data centers flexibly, for instance, by scheduling certain tasks, such as photo enhancement, to be executed in times of low overall energy demand. This is an example of where practice is ahead of research. While the flexibility of data centers has long been promoted, there is still little research on information systems that can help corporations to assess their ability of switching to real-time carbon-free energy supply. This is a major field of research for Information Systems scholars focused on sustainability (Dedrick 2010).

## Avenues for Green IS

Besides the corporate use of carbon-free energy, there are multiple areas in which Information Systems research can contribute to a more sustainable energy system. This includes gamification and nudges to promote the use of green electricity or sustainable energy products (Henkel et al. 2019), competitive gaming platforms for the design of innovative solutions (Ketter et al. 2016), charging coordination of electric vehicles and electric vehicle fleets to increase the share of renewable energy used (Flath et al. 2012), or data-based systems that recommend sustainable product alternatives (Tomkins et al. 2018). It includes the development and evaluation of algorithms, optimization methods, artificial intelligence, and decision support systems.

Our intention with this special issue is to provide a scientific outlet for implementable solutions that are capable of reducing carbon emissions instantly in the spirit of Gholami et al. (2016). We asked researchers to submit empirical findings or describe proto-systems that would directly enable practitioners to reduce carbon emissions.

Green IS has not yet made it to the center of attention for IS scholars despite the fact that climate change is likely to be the greatest challenge of the international community (Gholami et al. 2016). Five roadblocks for the academic community have previously been identified by Gholami et al. (2016) that keep Information Systems scholars from engaging in more impactful research regarding climate change. These are incentives misalignment, low status of practice science, data analysis poverty, identification of the proper research scope, and research method. In this editorial, we briefly revisit these issues and discuss what has changed in the past few years as well as proposing new ideas for the Information Systems community to drive more impactful and societally relevant research.

## Persistent Impediments

The misalignment of incentives has not dramatically shifted. It is still a gamble for young researchers to engage in implementable, practical research with a high ecological impact as this jeopardizes their prospective academic success. This results in young academics pursuing research that has high publication prospects, because the IS field does not recognize the dramatic need for practical climate change mitigation research. Once embedded in a research stream, it is often easier to stay with the flow and pursue research that is publishable. In order to change, we need to consistently provide outlets, both in the form of corresponding conference tracks and special issues that call for practical research in the field of Green IS. The coming ICIS 2021 in Houston certainly provides an opportunity with its conference theme of *Building Sustainability and Resilience with IS: A Call for Action*. However, in terms of funding, it is perceivable at least in the European Union and with the US Biden Administration that impactful projects will be favored over academic endeavors that do not generate knowledge that is applicable to solving the world’s major existential threat.

The low status of practice science with a focus on purposeful interventions (Seidel and Watson 2020) has changed somewhat in the sense that the importance of design science research has increased (Hevner et al. 2019). However, not every contribution can ultimately be translated into design theories or needs to be, which makes it more difficult to publish in high prestige outlets.

Today, there is an abundance of data available regarding the demand for energy, the supply of sustainable energy, and market data that describes the interaction between the two (e.g., Hirth et al. 2018). The Open Science community has greatly contributed to the field of energy (e.g., Brown et al. 2017). One of the papers in this special issue shows how we can use large data sets to generate empirical knowledge that can be put to practical use for reducing carbon emissions.

Interdisciplinary research, especially in the area of Green IS, seems more and more common. Many scholars publishing in the field of Green IS are also known to equally contribute to computer science-oriented conferences (e.g., Weigert et al. 2020; Weinhardt et al. 2019) and journals with a stronger (software) engineering perspective (e.g., Babic et al. 2017; Gust et al. 2016; Schuller et al. 2014). In addition, computer scientists are recognizing the importance of the energy system in the global context and create new outlets for energy-related research. The ACM eEnergy has been the flagship conference of energy informatics for years and the community has recently established the ACM SIG Energy^1^, which shows the recognition for energy research as a relevant field within computer science research. The two papers presented in this special issue both originate from research groups that work across disciplines. This is a trend that has not yet spread internationally and is rather focused on a few groups mostly in Europe. However, this development seems promising and can hopefully contribute to a stronger stance of Green IS in the Information Systems community.

Finally, regarding the research method, the associated need for theoretical contributions is still prevalent in the IS community. In his paper, “An Unhealthy Obsession with Theory” (Dennis 2019), Alan quotes a former colleague expressing the view that “IS research in top journals has shifted to doing autopsies on technologies that have been dead a few years” (p. 1407). This makes it difficult to publish applicable knowledge on innovative new products or ideas in IS as these cannot be integrated into a theory right away but rather need to be understood empirically before any further steps can occur. This focus on theory is further passed down to junior scholars who often review for IS conferences. They then discourage new doctoral students by questioning their theoretical contribution in empirical papers. One has to wonder what it means for a discipline if each and every paper in a conference with several hundred accepted papers produces new insights into theory, but too few test whether these theories are valid. Theory is not an end, it is a means of identifying applicable interventions that solve societal problems. Another related issue is that we need to move faster. In their editorial, Ågerfalk et al. (2020) note that review processes in the IS community are often too time consuming to make relevant contributions quickly as they compare the IS discipline against medical research during the Covid-19 pandemic. They point out that “Desperate times call for desperate measures.” (p. 205), which is equally true for finding solutions to the ongoing climate crisis.

Energy used to be a commodity with little emotional meaning. This has dramatically changed for some consumers. The energy sector is also relatively late to be digitalized. New solutions require case studies, initial field experiments, and simulations. These can often only be conducted with few participants or simplified versions of complex situations. All of this makes it more difficult to work through to actual theoretical insights. For this special issue, we received 10 submissions. We would have liked to publish more, but even though we received other high-quality submissions, these often did not meet the requirement of providing value for immediate practical implementation. This shows that the Green IS community is still small scale and not perceived as a promising avenue for tenure track appointments.

## A Way Forward

A quick search in the AISel library shows that at the past two ICISs about 10 review papers were accepted. For the past two ECISs, about 26 review papers were accepted. This is well and important as we need to condense our research results from time to time. However, it would be valuable for IS conferences and for the disciplines to also give room to an appropriate number of more practical papers, including and beyond design science, that provide initial insights through simulations or prototypes with small evaluations. Furthermore, we need to be more active in providing high quality outlets for applicable Green IS research. This ought to be driven by journal editors and conference chairs who need to acknowledge their responsibility for encouraging young academics to tackle problems that matter. The two accepted papers are good examples of how Green IS can contribute. The featured interview gives an impression of how interdisciplinary teams drive AI in utility companies which work at the interface of the Information Systems community, and how such systems need to be embedded in a strategy to reduce carbon emissions.

In *Benchmarking Energy Quantification Methods to Predict Heating Energy Performance of Residential Buildings in Germany*, the authors show how empirical research can directly translate into actionable knowledge. As the building sector is an often overseen driver of carbon emissions, they design a method that helps professionals to identify buildings with a high potential for retrofitting measures more quickly and without intensive auditing. They use a large data set to design a machine learning based model, and the results show that it might even outperform the traditional, more cumbersome approach. This model can directly contribute to lowering emissions by allowing state officials to classify a large number of buildings with regard to their potential for retrofitting and to design apt subsidy schemes.

In the second paper, *Not All Doom and Gloom: How Energy-Intensive and Temporally Flexible Data Center Applications May Actually Promote Renewable Energy Sources*, the authors design solutions that are at the forefront of current corporate energy policy as shown by Google’s 24/7 initiative. They evaluate the combination of server infrastructure that is operated using grid-supplied electricity with data centers that are directly connected to an intermittent renewable energy source. They show that for their use cases of Amazon Web Service requests and blockchain mining, the combination of a data center with a renewable generation unit is more profitable. This is a combination of Green IS and Green IT that contributes to the body of knowledge on data center flexibility and shows operators of such facilities how to improve their carbon footprints as well as their profits.

We hope that this special issue is of interest to you and that you agree with us and the reviewers that these papers show the tangible contribution that the Information Systems community can make to the global challenge of climate change.

## Footnotes

1 https://energy.acm.org/.
